# Deep Endometriosis Induced Spontaneous Colon Rectal Perforation in Pregnancy: Laparoscopy Is Advanced Tool to Confirm Diagnosis

**DOI:** 10.1155/2014/907150

**Published:** 2014-08-05

**Authors:** Aurelio Costa, Annalisa Sartini, Silvia Garibaldi, Marco Cencini

**Affiliations:** ^1^General Surgery Division, Ospedali Riuniti Valdichiana Senese Hospital, Montepulciano, 53040 Siena, Italy; ^2^Obstetrics and Gynecology Division, Ospedali Riuniti Valdichiana Senese, Via Provinciale No. 5, Località Nottola, Montepulciano, 53040 Siena, Italy

## Abstract

Endometriosis causes rare complications in pregnancy, such as obstetrical bleeding, preterm birth, spontaneous haemoperitoneum, and intestinal perforation. The prevalence of spontaneous perforation due to intestinal endometriosis is unknown in pregnancy. A recent review of the literature indicated 15 bowel complications caused by endometriosis during pregnancy or at the immediate postpartum period. The diagnosis of a bowel perforation can be difficult and in all of the cases reported necessitates an exploratory laparotomy. Anyway, in the majority of cases bowel perforation is not diagnosed during this laparotomy, and a repeat laparotomy is needed. Laparoscopy is being used increasingly in the diagnosis and operative management of acute abdomen. Laparoscopy can be a useful means of diagnosis and in addition a therapeutic tool in selected pregnant patients with abdominal pain. We report the first case of a pregnant woman with spontaneous double sigmoid and rectal perforation from decidualized endometriosis diagnosed by laparoscopy.

## 1. Background 

Endometriosis is characterized by benign proliferation of ectopic endometrial glands and stroma in the peritoneal cavity, resulting in inflammation and scarring, often leading to pelvic pain and infertility [1]. It affects 2%–10% of women of reproductive age [2]. We suppose that endometriosis generally regresses during pregnancy [3]. Anyway, endometriosis can cause rare complications in pregnancy, such as obstetrical bleeding, preterm birth, spontaneous haemoperitoneum, and intestinal perforation [4]. The diagnosis of a bowel perforation can be difficult and in all of cases reported necessitates an exploratory laparotomy [5]. Laparoscopy is being used increasingly in the diagnosis and operative management of acute abdomen. Laparoscopy can be a useful method of diagnosis and in addition a therapeutic tool in selected pregnant patients with abdominal pain. We report the case of a pregnant woman with spontaneous double sigmoid and rectal perforation from decidualized endometriosis diagnosed by laparoscopy. It also shows the possibility of surgical treatment without cesarean delivery at that time.

## 2. Case Presentation

**Figure 1:**
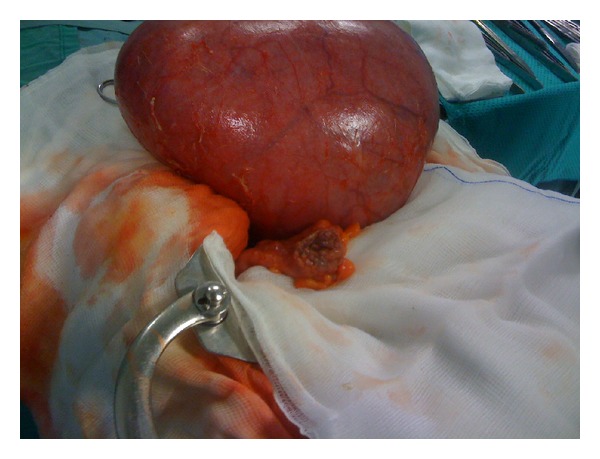
Intraoperative image.

A healthy 32-year-old Caucasian woman was admitted to emergency room with acute diffuse abdominal and pelvic pain, started 4 hours previously, at 25 weeks of pregnancy. She had an uncomplicated pregnancy. She had no reported history of endometriosis and previous abdominal surgery. Physical examination led to a diagnosis of pelvic peritonitis. Upon admission, temperature was 37.7°C and blood pressure was 110/62 mmHg. Laboratory tests revealed a white blood cell count of 18.500/mL, hemoglobin of 10.4 g/dL, and C-reactive protein (CRP) of 4.02 mg/dL (normal, 0.2 mg/dL). Abdominal ultrasound revealed minimal fluid around the liver, but neither air nor gross fluid was visible in the abdominal cavity. The obstetric evaluation ruled out an obstetric aetiology of patient's symptoms. The patient underwent an emergency operation for a presumptive diagnosis of acute diffuse peritonitis. Explorative laparoscopy was useful to confirm the clinical suspicion and revealed diffuse severe purulent peritonitis that originated from a double rectal and sigmoid perforation <1 cm in diameter related to an endometriotic nodule, that was confirmed in histopathological findings. A previous adherence, caused by endometriotic nodule, and the progressive traction of the enlarged uterus on the strictly adherent sigmoid colon could be a possible aetiopathogenetic mechanism. The surgical approach, for reduced intraperitoneal operative space, was continued through a median laparotomy. Purulent fluid was found in the abdominal cavity, and with further investigation a left sided sigmoid perforation was found associated with another perforation in rectosigmoid junction that was closed with linear stapler and sigmoid in site of perforation was chosen as stoma in left lateral abdominal wall. The patient tolerated the procedure well (Figure 1). The fetal status remained satisfactory and the patient's postoperative course was uneventful.* Escherichia coli, Enterobacter cloacae, and Enterococcus faecalis* were detected in the intraperitoneal fluid culture. Patient was treated with Meropenem 1 gr i.v. every 8 hours for 10 days and she was discharged on postoperative day 12 without any other complications. Physiological fetal growth and an uneventful antenatal period were reported until 41 weeks of gestation when a cesarean section was performed for unsuccessful vaginal delivery induction. The patient delivered a healthy female baby weighing 3750 gr with Apgar scores of 8 and 9 at one and five minutes, respectively. Colostomy was closed after 3 months without any complications.

## 3. Discussion

The incidence of acute abdomen during pregnancy is 1 in 500–635 pregnancies [6, 7]. Despite advancements in medical technology, preoperative diagnosis of acute abdominal conditions is still inaccurate.

Bowel perforation linked to endometriosis is a rare cause of the acute abdomen during pregnancy; there are only small number of reported cases: 12 cases of intestinal perforation due to endometriosis that was not associated with pregnancy and 13 cases occurred in pregnancy or at the immediate postpartum period [8–19].

Previous surgery for deep pelvic endometriosis or IVF treatment seems to be associated with this complication [8–19].

In our case there are not risk factors that could predict bowel complications.

The pathophysiology of endometriosis induced bowel perforation is not clear. Perforation of the bowel (stercoral perforation) is often due to pressure necrosis from fecal masses. In July 2000, Maurer et al. proposed a set of diagnostic criteria to differentiate stercoral perforation from other causes of bowel perforation [20]. According to the criteria outlined, a true stercoral perforation can be defined as follows.

- Perforations must be round or ovoid >1 cm in diameter, and must be antimesenteric in location.
- Fecal masses must be present within either the colon or abdominal cavity.
- Pressure necrosis or ulcer and chronic inflammatory reaction around the perforation site must be present microscopically.
- All of the above must be featured in the absence of any other active colonic pathology.

In our case one of possible mechanisms for large bowel perforation was due to increased abdominal pressure and also was facilitated by the progressive traction of the enlarged uterus on the strictly adherent between sigmoid-colon-recto and endometriotic nodule and consequently evolved in double rectosigmoid perforations.

In addiction in pregnancy endometriotic nodule becomes decidualized with progressive reduction in size that can lead to perforation by weakening intestinal wall.

Although endometriosis improves during pregnancy the current report shows the potential occurrence of serious and unexpected complications of the disease.

Both the rareness of the perforation and the symptoms that are suggestive of pyelonephritis or diverticulitis may be misleading and delay the diagnosis.

The diagnosis of a bowel perforation in all of cases reported necessitates an exploratory laparotomy but in the majority of cases bowel perforation is not diagnosed during this laparotomy and a new laparotomy is needed [5].

Laparoscopy can be useful means of diagnosis and a therapeutic tool and should be considered instead of laparotomy in selected pregnant patients with abdominal pain.

We report the first case of a pregnant woman with spontaneous double sigmoid and rectal perforation from decidualized endometriosis diagnosed by laparoscopy without cesarean section delivery at that time.

Indeed, the appropriate management of these patients may be challenging and a good outcome is absolutely dependent on a multidisciplinary approach.
